# Suture tension band fixation reduces hardware complications in olecranon fractures: a comparative study

**DOI:** 10.1016/j.jseint.2026.101734

**Published:** 2026-05-15

**Authors:** Zaamin B. Hussain, Frank Vazquez, Anthony J. Dugarte, Musab Gulzar, Kevin Cuneo, Michael B. Gottschalk, Eric R. Wagner

**Affiliations:** a Department of Orthopaedics, Rush University, Chicago, IL, USA; b Department of Orthopaedics, Emory University School of Medicine, Atlanta, GA, USA; c Department of Orthopaedics, University of Florida Jacksonville, Jacksonville, FL, USA

**Keywords:** Elbow, Fracture, Olecranon, Suture, Tension band, Trauma

## Abstract

**Background:**

Olecranon fractures requiring open reduction with internal fixation commonly undergo either plate fixation or tension band wire fixation. We describe surgical and post-operative functional outcomes after a novel variation of the tension banding technique utilizing Kirschner wires (K-wires) with eyelet holes and suture, compared to those that underwent plate fixation and tension band wire fixation. We hypothesized that suture tape tension banding would demonstrate noninferior surgical and functional outcomes.

**Methods:**

A retrospective review was conducted of all patients with Mayo IIA olecranon fractures who underwent operative transverse olecranon fracture fixation at a single institution between July 2017 and October 2023 with plate fixation, suture tape tension band fixation, or wire tension band fixation. Intraoperative data, patient-reported outcome measures, and functional outcomes were collected. Radiographs taken at least 12 weeks post-operatively were interpreted by the senior author.

**Results:**

In total, 43 patients underwent an open reduction with internal fixation for isolated olecranon fractures: 19 plate fixations, 16 suture tension band (STB), and 8 wire tension bands. The STB cohort had significantly shorter surgery times (68.2 min ±13) compared to the plate fixation (86.6 ± 19) and tension band with wire (88.0 ± 19) cohorts (*P* = .005). There was no difference in pain, functional outcomes, or range of motion among the 3 cohorts. Radiographs demonstrated 100% union rates for all patients in each cohort. There were no complications in the plate or STB groups, while 1 patient in the wire tension band had symptomatic hardware that required removal.

**Conclusion:**

This paper reports on a novel technique of STB using K-wires with eyelets. This technique appears to be associated with comparable outcomes to the 2 traditional techniques, including plate fixation and tension band fixation with K-wires in this preliminary study. However, it also required less overall surgical time. Future studies should focus on the long-term outcomes of this technique, as compared to traditional and more established techniques to treat olecranon fractures.

Olecranon fractures are the most common injury to the proximal ulna and account for 18% of proximal forearm fractures.^7^^,^^12^ This injury to the elbow extensor mechanism integrity causes disabling arm function. Treatment is challenging because of the highly deforming force of the triceps.^10^ When displaced, ideal treatment of these intra-articular fractures would provide anatomic alignment with rigid fixation, allowing early mobilization to prevent elbow stiffness.^20^ Displaced fractures with a stable ulnohumeral joint are the most common type, and when noncomminuted (Mayo Type IIA), surgical options include tension band wiring (TBW), intramedullary screw, and plate osteosynthesis.^10^

Numerous techniques for open reduction with internal fixation (ORIF) of olecranon fractures have been described but are associated with a high rate of reoperation and wound problems due to hardware prominence and irritation.^3^ Furthermore, a second surgery is frequently required with TBW that utilizes surgical steel wire, although long-term outcome data and low cost remain appealing.^17^ Precontoured olecranon locking plates (LPs) were subsequently developed to provide more rigid fixation, but they are also plagued by high rates of hardware removal from symptomatic hardware, as well as higher cost.^14^ As such, there is no widespread surgical option with high biomechanical stability using very low-profile implants while carrying a much lower risk of reoperation and return-to-operating room costs. All-suture olecranon tension band constructs have emerged as a low-profile option, but concerns remain about its ability to resist high tensile loads imparted by the triceps,^10^ and there is a paucity of clinical outcomes available.^8^

Kirschner wires with eyelets have been used with wire tension bands,^13^ but there is very little known on the outcomes of this technique. We describe a novel technique and early outcomes of fixation of simple olecranon fractures using a suture tension band (STB) with eyelet pins. We hypothesize that this technique will have equivalent short-term outcomes to LP and TBW fixation with lower rates of hardware removal and operative time.

## Materials and methods

A search was conducted following approval of the institutional review board, for patients who underwent an olecranon ORIF at our institution from July 2017 to March 2024. Only patients with isolated Mayo IIA olecranon fractures were included in our analysis. In addition, patients with prior surgical history in the injured elbow were excluded. Patients with less than 75 days of follow-up were excluded from post-operative outcomes analysis but were included for demographic and intraoperative analysis. Patients were grouped based on fixation type. Choice of fixation was determined by the treating surgeon based on preference. Those that underwent the STB fixation with eyelet pins were compared to the control groups of the LP and wire tension band (WTB) fixation.

### Data collection

Chart review was performed retrospectively for patients who met inclusion criteria. Information pertaining to patient demographics, intraoperative, and post-operative data was collected. Patient age, sex, body mass index, smoking status, and diabetes diagnosis were recorded for demographic data. Intraoperative data included surgical time and method of fixation. Patient-reported outcome measures (PROMs), such as Quick Disabilities of the Arm, Shoulder and Hand, Mayo Elbow Performance Score, Shoulder Subjective Value, and visual analog scale pain score, were recorded for post-operative outcomes analysis. Range of motion (ROM) measures recorded included elbow flexion, extension, pronation, and supination. Radiographic images at least 75 days post-operative were evaluated by the senior author to assess fracture healing. Patients who did not meet the minimum follow-up criteria were contacted prospectively to obtain data.

### Statistical analysis

Demographic data and surgery time were compared among all patients who underwent plate fixation, wire tension band fixation, and STB fixation for analysis. Group comparison analysis was also conducted for post-operative measures for patients who met the minimum follow-up. SPSS software (version 29.0, Sep. 2022; Armonk, NY, USA) was used for statistical analysis. An analysis of variance test was used to compare means of continuous variables, and a chi-square test was used to compare percentages of categorical variables. A value of *P* < .05 was used to assess statistical significance.

### Surgical technique: suture tension band

**Figure 1:**
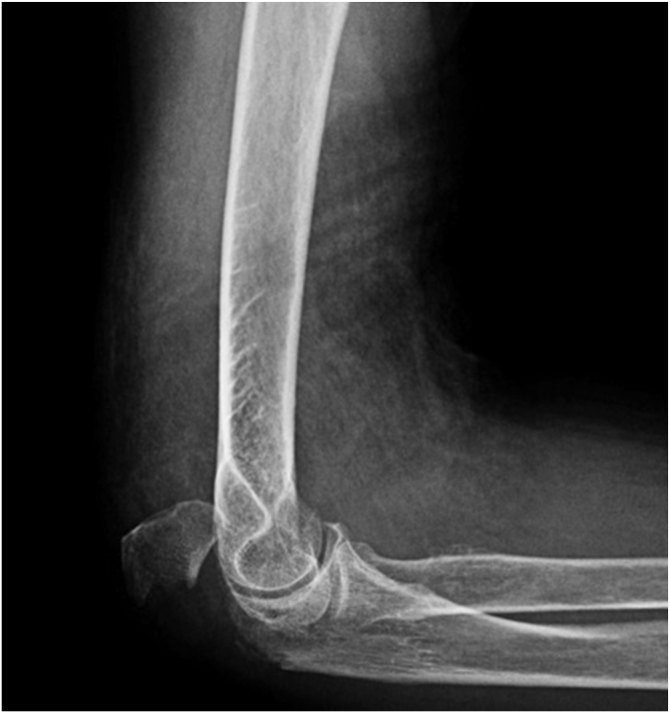
Radiograph of an 82-year-old female with a displaced Mayo type IIa olecranon fracture.

This technique is indicated for Mayo^18^ type IIa and some IIb olecranon fractures (Fig. 1), and has been described previously (Table I).^16^ The patient is positioned in lateral decubitus. The operative arm is draped over a radiolucent bolster. A 15- to 20-cm posterior incision is made, starting 5 cm distal to the tip of the olecranon and curving radially around it. Medial and lateral subcutaneous flaps are developed and the anconeus and extensor carpi ulnaris and flexor carpi ulnaris are elevated off the ulnar shaft to expose and débride interposed hematoma and soft tissue. The triceps tendon is then exposed approximately 8 to 10 cm.

**Table I:** Demographic characteristics.

Variable	Suture tension band (n = 18)	Plate (n = 22)	Wire tension (n = 8)	Overall (n = 48)	*P* value
Age	61.8 ± 21.0	55.5 ± 16.1	54.75 ± 18.9	57.71 ± 18.4	.502
Sex					1.00
Male	6 (33)	7 (32)	3 (38)	16 (33)	
Female	12 (66)	15 (68)	5 (62)	32 (66)	
BMI	24.7 ± 5.1	25.5 ± 4.6	23.0 ± 2.6	24.8 ± 4.6	.429
Smoking status					.765
Current	2 (11)	4 (18)	1 (13)		
Former	1 (6)	4 (18)	1 (12)		
Never	15 (83)	14 (64)	6 (75)		
Diabetic	1 (0.1)	0 (0)	0 (0)		.532

*BMI*, body mass index.

Data are presented as mean ± standard deviation or number (percentage).

**Figure 2:**
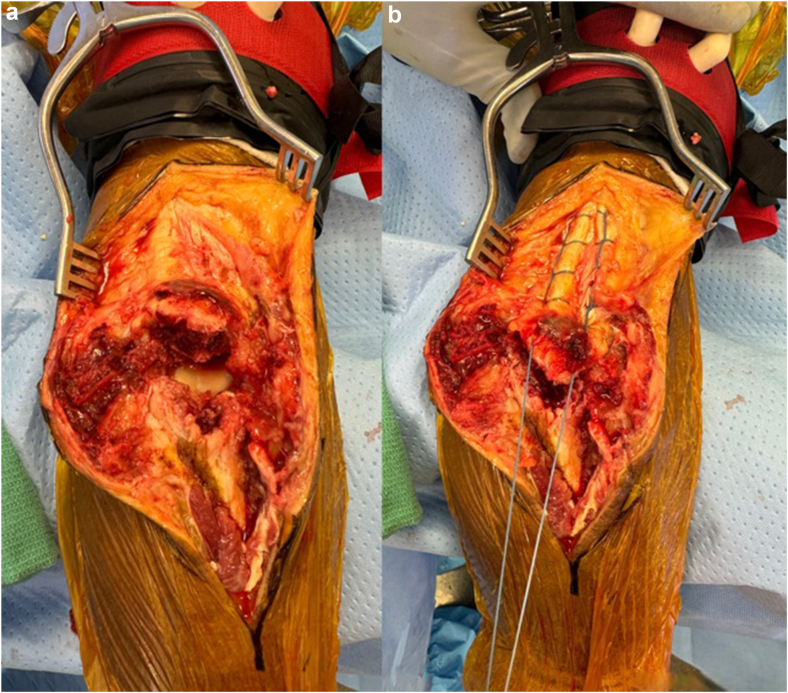
Right arm demonstrating the (**a**) posterior approach to the olecranon, exposing approximately 8-10 cm of triceps tendon and proximal ulna. (**b**) A #2 FiberWire is used to Krackow the triceps tendon.

A #2 FiberWire (Arthrex, Naples FL) suture is used in a locking Krakow configuration in the triceps tendon, leaving 2 free tails distally at its insertion (Fig. 2). Two 2.0-mm drill holes are placed from ulnar to radial in the ulnar shaft at 3 cm and 5 cm distal to the fracture, respectively. Next, a large pointed reduction clamp is used to reduce the fracture.

**Figure 3:**
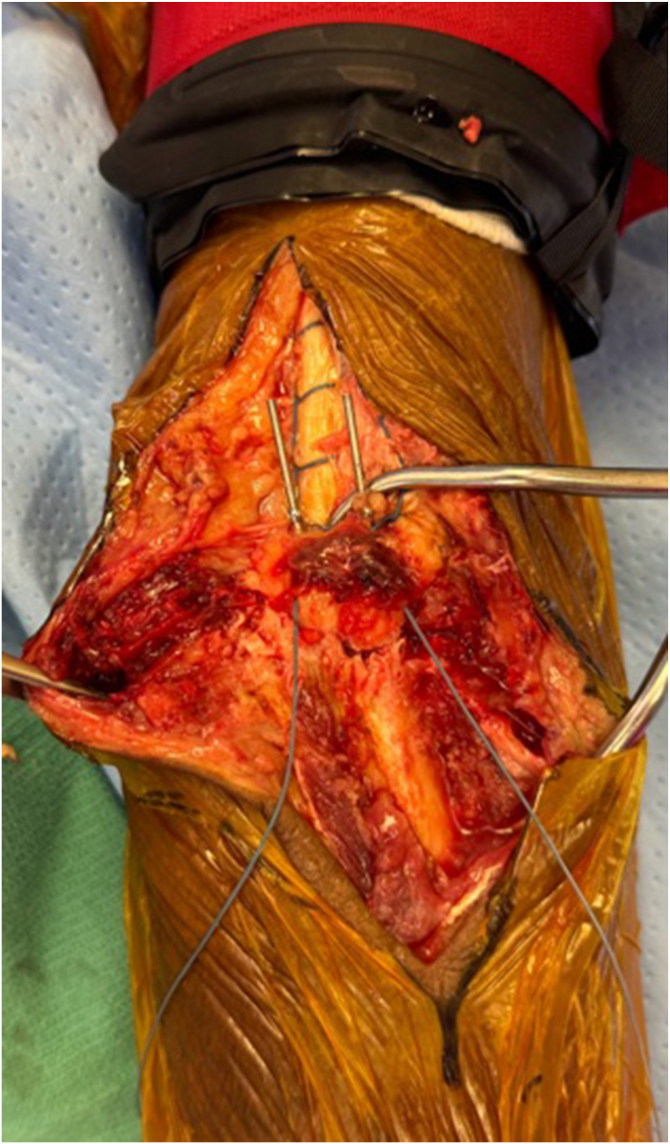
The fracture is reduced with a large pointed reduction clamp, and both eyelet wires are advanced across the fracture site under the guidance of multiplanar fluoroscopy.

**Figure 4:**
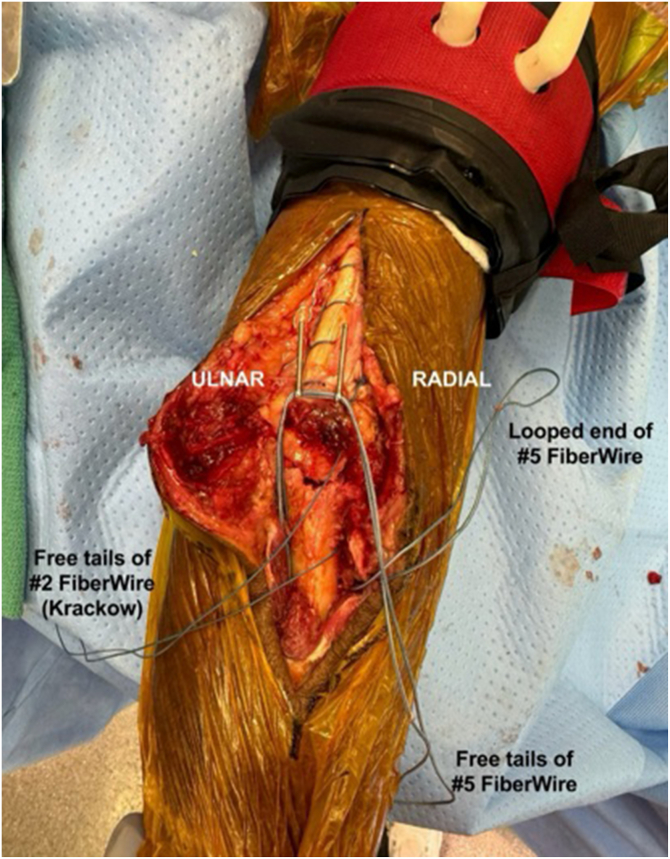
Right arm demonstrating the final position of all suture limbs before tying. All suture ends are on the radial side to avoid ulnar-sided knots, which may lead to ulnar nerve irritation.

Two eyelet wires are passed antegrade from the tip of the olecranon down the canal of the ulna, radially and ulnarly (Fig. 3). These should be down the canal or coming out the anterior cortex of the ulna. A #5 FiberWire suture is passed twice from ulnar to radial through both of the eyelets, leaving a loop on the ulnar side and 2 free tails on the radial side. The looped end is passed from through the distal drill hole, while the ulnar limb of the Krakow suture is passed through the proximal drill hole, positioning all knots on the radial side of the ulna to minimize ulnar nerve irritation (Fig. 4).

**Figure 5:**
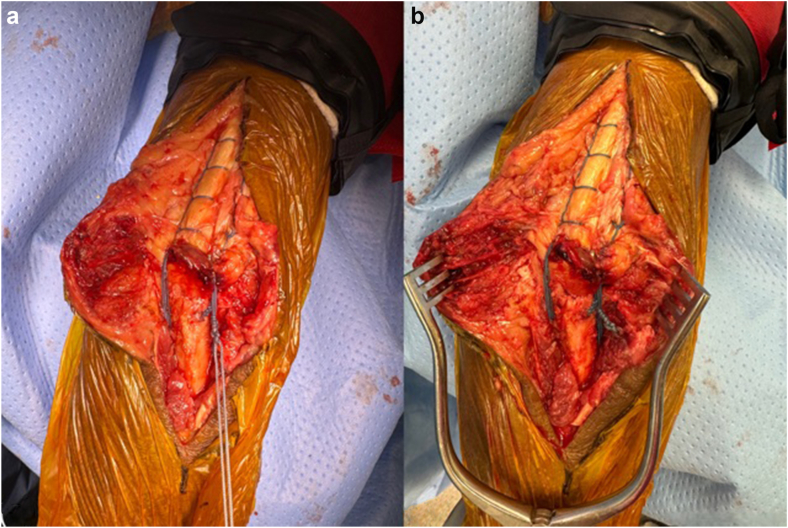
(**a**) Final construct after all sutures have been tied. (**b**) The elbow is taken through range of motion to ensure no gapping is present at the fracture site.

After incising the triceps tendon between the eyelet pins, the pins are tamped down, and the proud part of the pin outside the eyelet is broken off such that they are no longer prominent above the triceps tendon. The #5 FiberWire is tensioned, the #2 FiberWire is tied, and finally the #5 FiberWire is tied using a modified Nice knot (Fig. 5).

**Figure 6:**
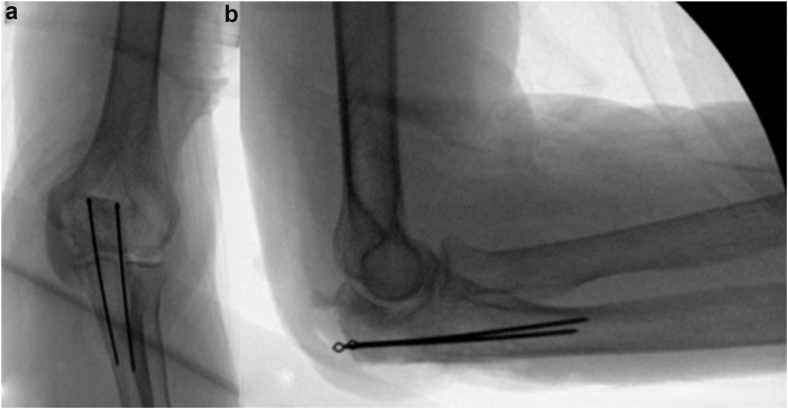
Post-operative radiographs of the final construct demonstrating appropriate reduction. (a) AP and (b) lateral radiographs.

The elbow is then ranged to confirm stability. Knots are buried deep to the extensor carpi ulnaris/flexor carpi ulnaris fascia, and the triceps tendon is closed with absorbable suture. Skin is closed with absorbable suture and skin glue. Radiographs are taken to confirm adequate final reduction (Fig. 6).

The patient is splinted in elbow flexion for 1 week. Active motion begins at 2 weeks, gentle weight-bearing at 8 weeks, and unrestricted activity at 3 months.

## Results

### Demographics

A total of 48 patients were included in our study, with 18 STB fixation, compared to 22 patients undergoing LP fixation and 8 undergoing WTB fixation. The overall mean age was 57.7 years, and there was no significant difference in age among the groups. Females made up most of the study population at 66%. Mean follow-up for all groups was 24.2 months (Table II).

**Table II:** Patient-reported outcome measures.

Variable	Suture tension band (n = 16)	Plate (n = 19)	Wire tension (n = 6)	Overall (n = 34)	*P* value
QDASH	17.6 ± 24.1	10.5 ± 10.8	11.4 ± 21.6	13.3	.536
Mayo Elbow	85.7 ± 20.3	89.7 ± 10.2	96.7 ± 14.9	89.2 ± 14.9	.313
VAS	1.06 ± 1.73	1.26 ± 1.94	0.50 ± .84	1.07 ± 1.72	.65
SSV	84.6 ± 17.7	86.3 ± 15.1	96.8 ± 5.9	87.2 ± 15.5	.25
Mean follow-up; mo	15.3 (3.7-35.4)	23 (4.7-63)	50.7 (38.9-64.3)	24.2 (3.7-64.3)	**<.001**

*QDASH*, Quick Disabilities of the Arm, Shoulder and Hand; *VAS*, visual analog scale; *SSV*, Shoulder Subjective Value.

Data are presented as mean ± standard deviation or mean (range).

Boldface represents statistically significant value.

### Outcomes

PROMs past 12 weeks of follow-up showed no significant differences among the 3 olecranon fixation modal**i**ties and the 4 PROMs (Table II).

### Range of motion

Differences in ROM were not significant among treatment groups for all 4 movements at the elbow joint (Table III).

**Table III:** Range of motion.

Variable	Suture tension band (n = 15)	Plate (n = 18)	Wire tension (n = 7)	Overall	*P* value
Flexion	136 ± 15	136 ± 12	138 ± 7	136 ± 14	.938
Extension	5 ± 7	7 ± 10	1 ± 2	5 ± 7	.190
Pronation	84 ± 9	87 ± 5	86 ± 7	86 ± 7	.473
Supination	79 ± 15	84 ± 8	85 ± 7	82 ± 11	.334
Mean follow-up; mo	15.4 (3.7-35.4)	21.2 (2.9-63.0)	43.8 (2.7-64.3)	22.7 (2.7-64.3)	**.003**

Data are presented as mean ± standard deviation or mean (range).

### Radiographic outcomes

When analyzing radiographs of patients, all 33 patients with available radiographs healed their fractures without any nonunions or delayed unions. There were no significant differences in time to union (Table IV).

**Table IV:** >12 weeks X-rays.

Variable	Suture tension band (n = 11)	Plate (n = 17)	Wire tension (n = 5)	Overall	*P* value
Healed	11	17	5	33	
Healing	-	-	-		
Mean time to union; mo	4.3 (2.7-7.6)	4.3 (2.5-12.2)	4.0 (2.7-6.0)	4.3 (2.5-12.2)	.081

Data are presented as number or mean (range).

### Complications

One patient in the WTB fixation group underwent removal of hardware 5 months post-operatively due to prominence of one of the pins. There were no other complications in any of the cohorts.

### Surgical factors

The overall mean surgery time was 79.1 minutes. LP had a mean surgery time of 84.4 minutes, STB was 68.3 minutes, and WTB was 88.0 minutes (*P* = .005) (Table V). LP had a mean tourniquet time of 56.5 minutes, STB was 46 minutes, and WTB was 54 minutes (*P* = .262).

**Table V:** Surgical data.

Variable	Suture tension band (n = 18)	Plate (n = 22)	Wire tension (n = 8)	Overall (n = 48)	*P* value
Surgery time (min)	68.3 ± 12.5	84.4 ± 18.0	88 ± 18.7	79.1 ± 18.1	**.005**
Reoperations	0	0	1∗		

Data are presented as mean ± standard deviation or number.

Boldface represents statistically significant value.

∗ One tension band wire fixation patient underwent reoperation for removal of hardware due to hardware irritation.

## Discussion

Olecranon fractures are commonly managed operatively when displaced, with standard fixation options including LP and WTB. However, these constructs are associated with hardware-related complications and often require reoperation.^6^^,^^11^^,^^15^ Lower-profile constructs with sutures have the potential to improve upon these reoperation rates, with comparable outcomes in preliminary findings. This study assessed the clinical utility of a novel STB construct using eyelet pins, comparing it to LP and WTB.

Our findings showed that STB fixation resulted in significantly shorter surgical times compared to both LP and WTB techniques, with overall comparable clinical outcomes. There were no observed differences in PROMs or ROM, with a 100% rate of radiographic union and no complications among the 3 groups. The only reoperation was seen in the WTB group.

When examining post-operative function, patients across all groups achieved excellent ROM and reported favorable outcomes. Mean PROMs and elbow ROM were similar among the STB, LP, and WTB cohorts, with no statistically significant differences. These results are consistent with prior studies demonstrating that suture-based tension band constructs provide similar functional recovery to traditional fixation. In a retrospective series of displaced olecranon fractures treated with all-STB fixation, Vesterby et al^22^ reported median flexion and extension deficits of 0° and similarly low Quick Disabilities of the Arm, Shoulder and Hand scores, supporting the technique's ability to preserve motion while providing great outcomes in the patient. In a systematic review, Dogramatzis et al^5^ reported no difference in final elbow arc of motion between STB and WTB. Taken together, these findings suggest that suture-based constructs reliably restore motion and function, reinforcing their role as a safe and effective alternative for olecranon fracture fixation.^5^

While our results demonstrated low complication rates across all fixation groups, this is likely underdetection due to the relatively short follow-up period. Both WTB and LP are well documented to exhibit high rates of hardware irritation and removal, often necessitating a second surgery. Romero et al^17^ retrospectively evaluated WTB constructs and reported a 71.7% reoperation rate, the majority of which were due to hardware irritation. This rate varies considerably among publishes series, with other authors describing hardware removal rates less than half of that reported by Romero et al.^19^ LPs have a lower, through still substantial, rate of hardware irritation and removal. Reported rates of LP reoperation range from 22% to 42%.^1^^,^^14^^,^^19^ The difference among LP and WTB vs. STB is likely explained by the lower implant profile and compliant nature of the suture material, which reduces focal pressure points beneath the skin and prevents the soft-tissue abrasion commonly seen with stiff metal constructs. In addition, the absence of twisted wire ends or screw heads near the subcutaneous border further reduces the risk of local irritation. Long-term outcomes studies of this technique are needed to assess rates of hardware irritation and removal compared to other techniques.

While a formal cost analysis was not performed, this STB technique may be more cost-effective than LP fixation. At the senior author's institution implant and supply costs were roughly $700 for the suture fixation technique and roughly $2,400 for the LP technique. There could be additional cost savings from the reduced operating room time utilization, as there are no screws to measure and insert and no decisions to make about plate size. This is consistent with prior published cost analyses showing WTB to be significantly lower cost than LP fixation.^9^^,^^14^ Further research including a formal cost analysis can better define these differences.

Achieving good outcomes with this technique relies on appropriate patient selection. Tension band constructs function by converting tensile stresses at one cortex into compressive forces at the opposing cortex.^2^ In the case of olecranon fractures, the triceps exerts a proximal tensile force on the proximal fragment.^2^^,^^21^ A posterior tension band, either wire or suture, coverts this to a compressive force at the articular surface.^21^ This force coupling requires intact cortices; therefore, tension band constructs such as this technique would be appropriate for some simple patterns of Mayo IIB, but not the more comminuted ones.^4^^,^^10^ In addition, ulnohumeral instability (Mayo type III olecranon fractures) is a contraindication to this technique, and we use LP fixation for those cases.^4^^,^^10^

This study has several limitations. While early outcomes of this technique are promising, long-term studies are needed to fully understand outcomes and complication rates of this technique. Indeed, a lower minimum follow-up time frame allowed us to include more patients for the intraoperative analysis to reduce fragility; however, we acknowledge that the short-term follow-up remains a limitation. The sample size of each group was small, and this study is likely underpowered and at risk of Type II error. We had 69% radiographic follow-up, which represents real-world practice patterns in a trauma population, and indeed these missing data may introduce bias. This technique appears to produce excellent clinical results, but future biomechanical studies are warranted to compare fixation strength to established techniques. This study was retrospective at a single institution, and treatment allocation was based on surgeon preference, which could introduce selection bias and confounding. Future prospective randomized trials could better compare these techniques of olecranon fixation.

## Conclusion

This novel technique of STB fixation using eyelet Kirschner wires is a reliable option for ORIF of Mayo Type IIA olecranon fractures. Compared to traditional methods, such as WTB or LP, this STB technique significantly reduces surgical time, with comparable clinical outcomes in this preliminary study. The utilization of this STB method could potentially streamline surgical procedures without compromising the quality of the care. Future studies are needed to understand the long-term outcomes of this technique compared to the traditional ones.

## Declaration of generative AI and AI-assisted technologies in the writing process

During the preparation of this work the authors did not use AI for any reason. All authors agree to this statement.

## Disclaimers:

Funding: No funding was disclosed by the authors.

Conflicts of interest: Eric Wagner receives consulting fees from Stryker, Smith and Nephew, Depuy-Synthes, and Acumed. He receives institutional research support from Konica Minolta. He is a board or committee member of the American Society for Surgery of the Hand, American Shoulder and Elbow Surgeons, and American Academy of Orthopedic Surgeons. He is a deputy editor for the Journal of American Academy of Orthopedic Surgeons and Journal of Hand Surgery GO.

Michael B. Gottschalk, MD: This author receives institutional support from Skeletal Dynamics, Acumed, and Arthrex. He receives research support from Stryker and Konica Minolta. He is a board or committee member of the American Society for Surgery of the Hand. He is an associate editor for the *Journal of Hand Surgery* and *Surgical Techniques in Orthopedics*. He receives no royalties from any of the companies.

Any additional authors, their immediate families, and any research foundations with which they are affiliated have not received any financial payments or other benefits from any commercial entity related to the subject of this article.
